# Haplotype-resolved genome assembly of female *Vernicia montana* elucidates its W-specific sex-determination region

**DOI:** 10.1093/hr/uhag149

**Published:** 2026-04-16

**Authors:** Xiang Dong, Huaguo Zhu, Junyong Cheng, Lan Wu, Fei Zhang, Yuxin Liu, Xinxin Tang, Xinqi Cheng, Wenying Li, Lin Zhang

**Affiliations:** College of Biology and Agricultural Resources, Huanggang Normal University, Huanggang, Hubei 438000, China; Economic Crop Research Institute, Hubei Academy of Agricultural Sciences, Wuhan, Hubei 430000, China; College of Biology and Agricultural Resources, Huanggang Normal University, Huanggang, Hubei 438000, China; Hubei Key Laboratory of Economic Forest Germplasm Improvement and Resources Comprehensive Utilization, Huanggang, Hubei 438000, China; Hubei Academy of Forestry, Wuhan, Hubei 430000, China; College of Biology and Agricultural Resources, Huanggang Normal University, Huanggang, Hubei 438000, China; Hubei Key Laboratory of Economic Forest Germplasm Improvement and Resources Comprehensive Utilization, Huanggang, Hubei 438000, China; College of Biology and Agricultural Resources, Huanggang Normal University, Huanggang, Hubei 438000, China; Hubei Key Laboratory of Economic Forest Germplasm Improvement and Resources Comprehensive Utilization, Huanggang, Hubei 438000, China; College of Biology and Agricultural Resources, Huanggang Normal University, Huanggang, Hubei 438000, China; Hubei Key Laboratory of Economic Forest Germplasm Improvement and Resources Comprehensive Utilization, Huanggang, Hubei 438000, China; College of Biology and Agricultural Resources, Huanggang Normal University, Huanggang, Hubei 438000, China; Hubei Key Laboratory of Economic Forest Germplasm Improvement and Resources Comprehensive Utilization, Huanggang, Hubei 438000, China; College of Biology and Agricultural Resources, Huanggang Normal University, Huanggang, Hubei 438000, China; Hubei Key Laboratory of Economic Forest Germplasm Improvement and Resources Comprehensive Utilization, Huanggang, Hubei 438000, China; College of Biology and Agricultural Resources, Huanggang Normal University, Huanggang, Hubei 438000, China; Hubei Key Laboratory of Economic Forest Germplasm Improvement and Resources Comprehensive Utilization, Huanggang, Hubei 438000, China; State Key Laboratory of Woody Oil Resources Utilization, Central South University of Forestry and Technology, Changsha 410004, China

## Abstract

*Vernicia montana* is a dioecious woody tree with a ZW sex determination system, yet the molecular basis of sex determination remains poorly understood. Here, we constructed a chromosome-level, haplotype-resolved female genome assembly using PacBio HiFi sequencing and Hi-C scaffolding, and integrated population-scale coverage-based genome-wide association analysis of 178 natural individuals to identify sex-associated loci. A highly localized sex-associated signal was detected on ChrB02, defining a ~61.4-kb W-specific sex-determining region (SDR) that is sharply bounded by conserved syntenic blocks but internally enriched in haplotype-specific insertions, duplications, inversions, and a complex inverted-repeat architecture. Polymerase chain reaction assays further validated multiple female-specific segments within this structurally heterogeneous region. Two conserved genes were identified at the SDR boundaries. Notably, *VmBASS4.2* displayed pronounced developmental dynamics and broad reproductive expression and is positioned adjacent to major structural rearrangements within the SDR, making it a compelling candidate for functional validation. Heterologous overexpression in *Arabidopsis* impaired stigma receptivity and increased floral and silique abortion, supporting a dosage-sensitive role in reproductive development. Collectively, our findings suggest that sex determination in *V. montana* is not driven by the emergence of a novel sex-determining gene but instead is associated with local inverted-repeat-mediated structural remodeling that reshapes the regulatory landscape of pre-existing boundary genes such as *VmBASS4.2*. This study proposes an inverted-repeat-mediated SDR evolution model, providing a plausible framework linking local structural architecture to regulatory divergence during the early evolution of homomorphic sex chromosomes in plants.

## Introduction

Sex determination has evolved repeatedly across flowering plants and represents a striking example of developmental and genomic innovation [1, 2]. In dioecious species, male and female functions are typically controlled by sex-determining regions (SDRs), which often occupy restricted genomic intervals yet exert profound effects on reproductive development [3–5]. Understanding how such regions originate, diverge, and remodel regulatory networks is central to elucidating the evolutionary transition from hermaphroditism to separate sexes [6].

Recent advances in sequencing technologies and comparative genomics have rapidly expanded our understanding of sex determination in dioecious plants [4]. Several well-resolved systems have demonstrated that sex can be controlled by a small number of key genes embedded within compact SDRs. For example, in *Actinidia*, two Y-linked factors, *SyGI* and *FrBy*, act antagonistically to suppress female development and promote male function, respectively [7, 8]. In wild *Vitis* species, sterility-determining genes, including *VviINP1* and *VviYABBY3*, have been identified within the SDR, providing a molecular framework for the evolution of unisexual flowers [9–11]. Similarly, SDR structure and sex chromosome differentiation have been investigated in *Asparagus officinalis*, *Cannabis sativa*, *Spinacia oleracea*, and *Fragaria moupinensis* [12–16]. Notably, a conserved epigenetic mechanism centered on partial duplication and sex-specific methylation-mediated silencing of *ARR17* orthologs has been reported across Salicaceae, suggesting that recurrent evolutionary solutions may emerge despite the broad diversity of sex-determining systems [17–22].

Despite these advances, many plant SDRs remain difficult to resolve at the sequence level. SDRs are frequently enriched in structural variation, repeat accumulation, and haplotype-specific insertions or inversions, which can obscure collinearity and complicate the identification of causal genes [22–24]. Moreover, beyond the discovery of sex-linked markers, an important unresolved question is how structural remodeling within SDRs reshapes local regulatory landscapes and contributes to the stabilization of sex-specific developmental programs.

*Vernicia montana* provides an informative system for investigating these processes. *V. montana* is a dioecious woody oilseed tree native to China and is widely cultivated as a distinctive ornamental and landscape tree, valued for its elegant canopy architecture and its abundant, showy spring inflorescences [25, 26]. The species exhibits pronounced sexual dimorphism in floral morphology: female flowers retain a ring of aborted stamens, whereas male flowers completely lack pistils [27]. In addition, male and female flower buds display markedly different developmental timing, and sexual expression in *V. montana* appears relatively unstable, as occasional female flowers and fruit set can be observed on otherwise male individuals [27]. These features make *V. montana* a particularly intriguing system for exploring the genomic basis and evolutionary dynamics of sex determination.

In our previous studies, we generated a chromosome-level male reference genome and established that *V. montana* likely possesses a ZZ/ZW sex chromosome system [25]. We further mapped sex determination to a single InDel locus on chromosome 2, enabling the development of a robust molecular assay for early sex identification [25]. However, the genomic architecture of the SDR, the structural mechanisms driving haplotype divergence, and the regulatory remodeling associated with sex-linked differentiation remain largely unresolved.

In this study, we generated a chromosome-level, haplotype-resolved genome assembly of a female *V. montana* using PacBio HiFi sequencing and Hi-C scaffolding. By integrating population-scale resequencing, coverage-based association analyses, fine-scale structural variant detection, and experimental validation, we identify a highly differentiated W-specific SDR on ChrB02 characterized by pronounced loss of collinearity and complex inversion-associated repeat structures. We further prioritized genes located within the SDR and performed heterologous overexpression assays in *Arabidopsis thaliana*, providing functional evidence suggesting that altered expression of *VmBASS4.2* can perturb reproductive development. Together, these results illuminate how structural divergence coupled with regulatory remodeling may contribute to SDR evolution in a recently derived dioecious woody species.

## Results

### Genome assembly, annotation, and assessment

**Figure 1:**
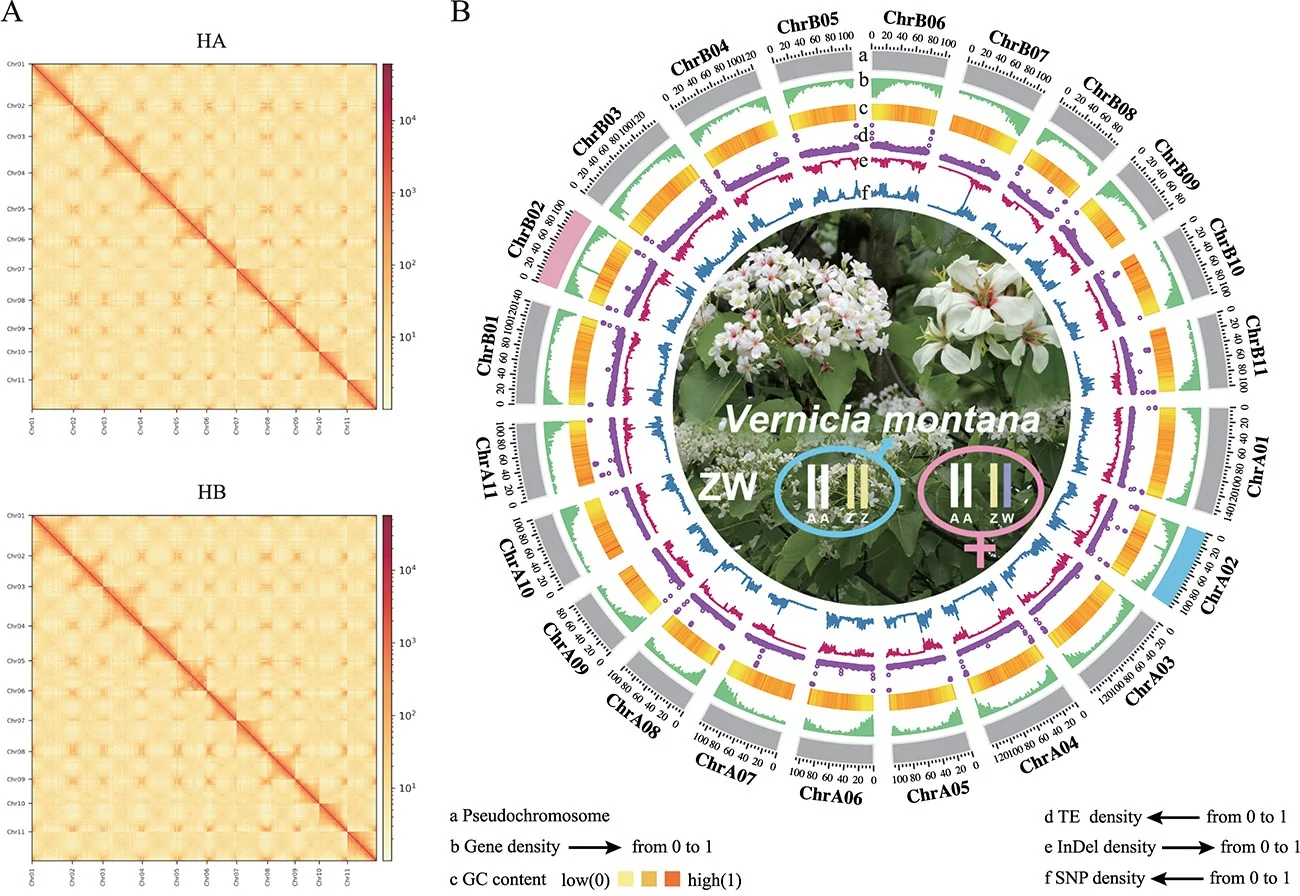
Overview of the haplotype-resolved genome assembly of *V. montana* (A) Hi-C interaction heatmaps at 500-kb resolution for HA and HB, showing chromatin interaction intensity across the two haplotypes. (B) Circular genome landscape of *V. montana*, depicting pseudochromosomes (a) and the genomic distribution of gene density (b), GC content (c), transposable element (TE) density (d), Insertion/Deletion (InDel) density (e), and single-nucleotide polymorphism (SNP) density (f).

A total of 31.96 Gb of PacBio HiFi data and ~137.96 Gb of Hi-C reads were generated from a female *V. montana* individual (2n = 2x = 22; Table S1). Based on k-mer frequency analysis, the haploid genome size of *V. montana* was estimated to be ~1.33 Gb, with a heterozygosity rate of 0.49% (Fig. S1). By integrating PacBio HiFi sequencing with Hi-C scaffolding (Table S1), two chromosome-level haplotype-resolved genome assemblies, designated haplotype A (HA) and haplotype B (HB), were successfully constructed (Fig. 1A). Approximately 98.56% of HA assembly and 98.91% of HB assembly were anchored to 11 pseudochromosomes (Table 1; Fig. 1B). The final assembled genome size was 1.25 Gb for HA, comprising 298 unanchored contigs not assigned to pseudochromosomes, and 1.24 Gb for HB, comprising 177 unanchored contigs (Table 1).

**Table 1:** Assembly statistics for the haplotype-resolved genomes of *V. montana.*

	**HA**	**HB**
Total length (bp)	1 251 375 937	1 238 100 088
Number of contigs	1332	984
Contig N50	6 045 548	5 384 806
Number of unanchored contigs	298	177
Total length of unanchored contigs (bp)	18 071 726	13 523 297
Anchor rate (%)	98.56%	98.91%
GC content	35%	35%
BUSCO completeness (%)	98.5%	98.6%
Complete and single-copy BUSCOs (%)	96.8%	97.3%

The quality and accuracy of the haplotype-resolved assemblies were comprehensively evaluated using Benchmarking Universal Single-Copy Ortholog (BUSCO), the LTR Assembly Index (LAI), and HiFi read remapping. The HA and HB assemblies contained 1562 (96.8%) and 1570 (97.3%) complete and single-copy BUSCOs from the embryophyta_odb10 dataset, respectively, with only 15 (HA) and 14 (HB) BUSCOs missing (Table 1 and Table  S2). Both assemblies achieved reference-standard LAI scores (16.43 for HA and 16.36 for HB; Fig. S2), indicating high continuity across repetitive regions. In addition, consensus quality values (QVs) estimated from HiFi read alignments reached 62.66 for HA and 62.63 for HB, indicating >99% base-level accuracy.

Gene structure annotation identified 28 984 protein-coding genes in the HA assembly, with an average gene length of 3630.64 bp, and 28 976 genes in the HB assembly, averaging 3618.60 bp (Table S3). Functional annotation was assigned to 91.36% of HA genes and 91.47% of HB genes (Table S4). BUSCO assessment of annotation completeness based on the Embryophyta dataset further confirmed high coverage, with 1564 (96.9%) and 1577 (97.7%) complete and single-copy BUSCOs identified in HA and HB, respectively (Table S5).

Repeat annotation, integrating *de novo* and homology-based approaches, identified 1 339 609 and 1 475 653 transposable elements (TEs) in the HA and HB assemblies, accounting for 81.13% and 80.63% of the respective genomes (Table S6). Long terminal repeat retrotransposons (LTR-RTs) constituted the dominant repeat class, with *Gypsy* elements being the most abundant, followed by *Copia* elements. Overall, LTR-RTs comprised 66.10% of the HA genome and 70.29% of the HB genome (Table S6). At the chromosomal level, TE content ranged from 76.57% (ChrA09) to 83.76% (ChrA04) in the HA assembly and from 76.88% (ChrB09) to 82.99% (ChrB04) in the HB assembly (Table S7).

Noncoding RNA annotation further enriched the genomic resources. In the HA assembly, 97 microRNAs (miRNAs), 669 transfer RNAs (tRNAs), 4754 ribosomal RNAs (rRNAs), and 256 small nuclear RNAs (snoRNAs) were predicted, whereas the HB assembly contained 98 miRNAs, 673 tRNAs, 4226 rRNAs, and 237 snoRNAs (Table S8).

Taken together, these results demonstrate that the haplotype-resolved *V. montana* genome assemblies exhibit high continuity, accuracy, and completeness, providing a robust foundation for downstream comparative genomics and functional studies.

### Comparative analysis of genome assembly and annotation between male and female *V. montana*

To assess the comparability of genome assemblies and annotations between male and female *V. montana*, we systematically compared metrics from the previously published male reference genome ^[25]^ and the newly generated haplotype-resolved female genome assemblies (HA and HB；Table S9). The male genome, assembled using PacBio CLR sequencing combined with Hi-C scaffolding, had a total size of 1.29 Gb, with 97.28% of the assembled sequences anchored to 11 pseudochromosomes [25]. In comparison, the female haplotype-resolved genome assemblies, generated using PacBio HiFi sequencing and Hi-C scaffolding, exhibit highly comparable genome sizes (1.25 Gb for HA and 1.24 Gb for HB), with anchoring rates exceeding 98.5%. These results indicate overall concordance in genome size and chromosomal organization between male and female individuals.

Gene annotation revealed similar numbers of predicted protein-coding genes across the assemblies, with 28 781 genes identified in the male genome and 28 984 and 28 976 genes annotated in the female HA and HB assemblies, respectively. Functional annotation coverage was likewise comparable, with 92.24% of male genes and 91.36% (HA) and 91.47 (HB) of female haplotype genes assigned putative functions. BUSCO analysis based on the Embryophyta dataset demonstrated high annotation completeness across all assemblies, with 94.3% complete BUSCOs in the male genome and 96.9% and 97.7% in the female HA and HB assemblies, respectively. The higher BUSCO completeness observed in the female assemblies likely reflects the increased accuracy of HiFi sequencing.

Despite differences in sequencing platforms and assembly strategies, repeat annotation revealed broadly similar TE landscapes between male and female genomes. TEs accounted for 78.58% of the male genome and 81.13% (HA) and 80.63 (HB) of the female haplotype genomes, with LTRs, particularly *Gypsy* and *Copia* elements, representing the dominant repeat classes in all assemblies. Notably, the haplotype-resolved female genomes exhibited improved assembly continuity, reflected by fewer contigs and reference-standard LAI scores, highlighting the advantages of HiFi sequencing for resolving highly repetitive regions.

Collectively, these results demonstrate that the male and female *V. montana* genome assemblies exhibit highly consistent annotation completeness, gene content, and repeat composition. The improved continuity and haplotype resolution of the female genome provide an enhanced framework for fine-scale comparative genomic analyses, particularly for the identification and characterization of sex-determining regions.

### Comparative synteny analysis between female haplotypes and the male *V. montana* genome

**Figure 2:**
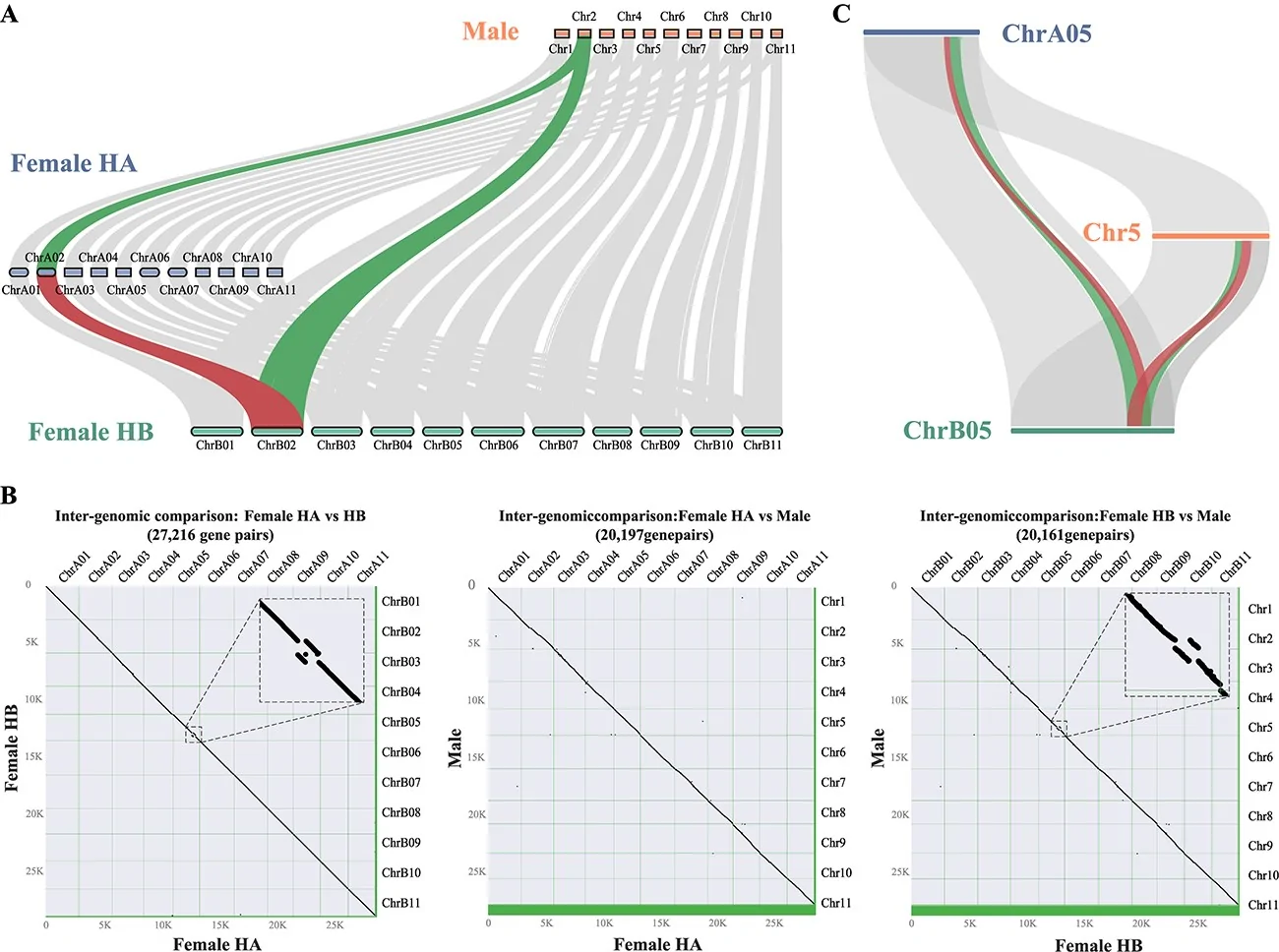
Synteny analyses reveal structural conservation and localized divergence between female haplotypes and the male reference genome. (A) Chromosome-level synteny karyotype illustrating collinear relationships among the two female haplotypes (HA and HB) and the male reference genome. (B) Dot-plot representations of intergenomic synteny comparisons between the two female haplotypes (HA and HB) and the male reference genome. Localized regions exhibiting apparent translocation signals on chromosome 5 in the HA vs. HB and HB vs. male comparisons are highlighted and enlarged. (C) Local synteny view focusing on orthologous regions of ChrA05 (HA), ChrB05 (HB), and Chr5 (male). Syntenic blocks highlighted indicate the translocated segments.

To evaluate large-scale structural conservation and divergence between male and female genomes, we performed pairwise synteny analyses among the two female haplotypes (HA and HB) and the published male reference genome^[25]^, using collinear gene pairs as anchors (Fig. 2). The comparison between HA and HB revealed nearly complete chromosome-level synteny and the highest number of collinear gene pairs, indicating strong structural conservation between the two haplotypes and further supporting the high quality of the haplotype-resolved assemblies (Fig. 2A).

By contrast, although overall chromosomal synteny was largely maintained in comparisons between each female haplotype and the male genome, both the HA-male and HB-male alignments exhibited a reduced number of syntenic gene pairs and several localized disruptions (Fig. 2B and C). These localized deviations suggest increased sequence divergence between male and female genomes, potentially reflecting sex-associated genomic differentiation. Notably, a translocation-like signal on chromosome 5 was consistently detected in the HA-HB and male-HB comparisons, whereas the corresponding region remained largely syntenic in the HA-male alignment (Fig. 2B and C). The recurrence of this signal across independent pairwise analyses supports its interpretation as a genuine haplotype-specific structural variant on chromosome 5, most likely associated with HB, rather than an assembly artifact or a genome-wide rearrangement.

Taken together, the strong synteny observed between HA and the male genome suggests that HA may retain a configuration closer to an ancestral or canonical state, whereas HB likely harbors a derived structural rearrangement.

### Allelic imbalance analysis

**Figure 3:**
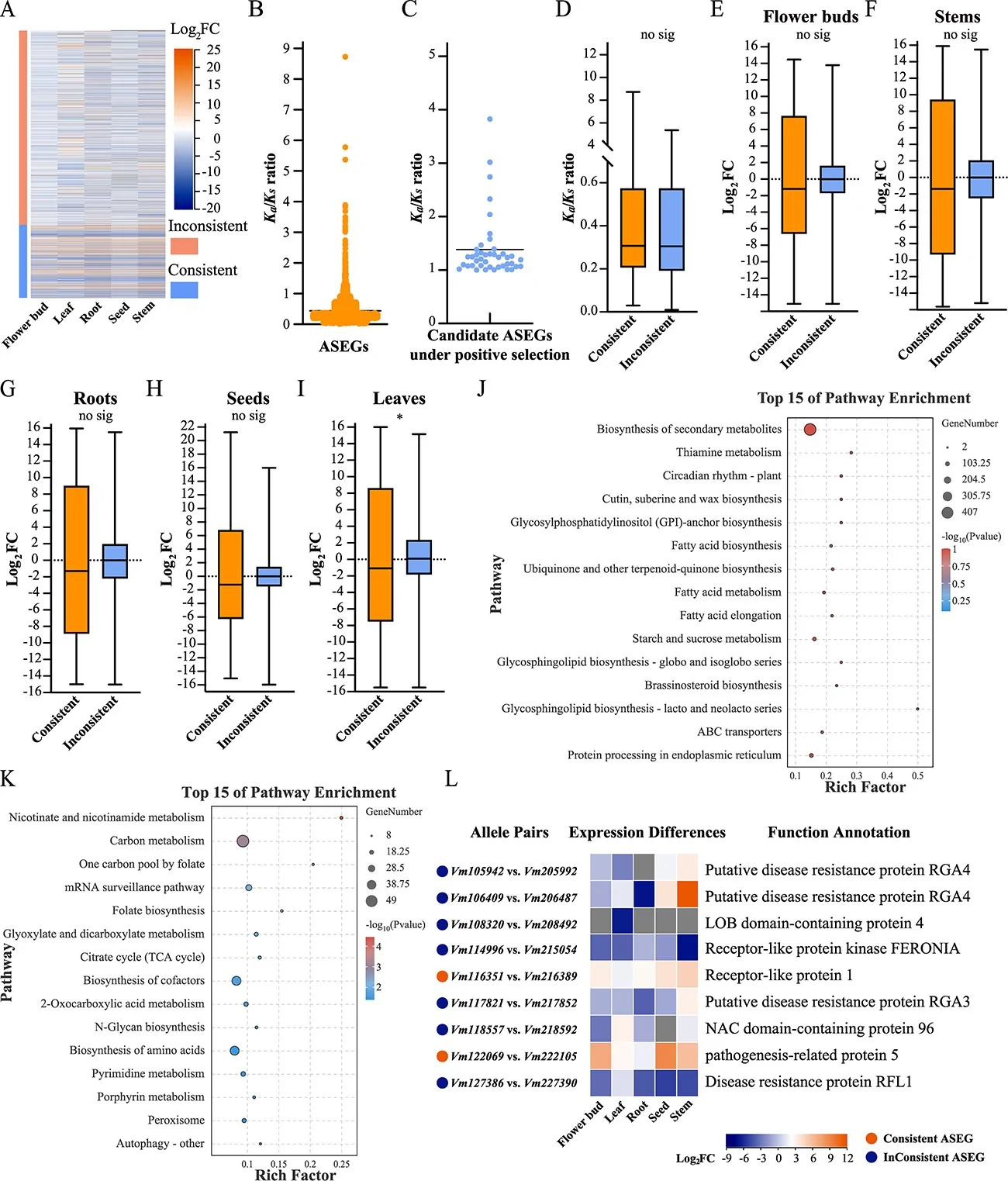
Allelic imbalance analysis of allele-specific expression genes (ASEGs). (A) Heatmap showing allelic expression differences (log₂ fold change, log₂FC) of ASEGs across five tissues, with genes classified as consistent or inconsistent ASEGs based on expression patterns. (B) Scatter plot showing the distribution of *K_a_*/*K_s_* ratios for all ASEGs. (C) Scatter plot of *K_a_*/*K_s_* ratios for candidate ASEGs under positive selection (*K_a_*/*K_s_* > 1). (D) Comparison of *K_a_*/*K_s_* ratios between consistent and inconsistent ASEGs. (E–I) Boxplots comparing allelic expression differences (log₂FC) between consistent and inconsistent ASEGs in flower buds (E), stems (F), roots (G), seeds (H), and leaves (I). Statistical significance is indicated where applicable. (J) KEGG pathway enrichment analysis of inconsistent ASEGs. (K) KEGG pathway enrichment analysis of consistent ASEGs. (L) Heatmap showing allelic expression divergence (log₂FC) of representative candidate ASEGs under positive selection across five tissues, with functional annotations indicated.

Allelic imbalance analysis was conducted to characterize allele-specific expression (ASE) patterns across multiple tissues. A total of 26 899 allele pairs were identified between the two haplotypes. ASE analysis across flower buds, stems, roots, leaves, and seeds detected significant allelic imbalance (FDR < 0.05) in 6745 (25.08%), 5961 (22.16%), 5726 (21.29%), 4995 (18.57%), and 4962 (18.45%) genes, respectively. In total, 9141 genes exhibited significant ASE in at least one tissue and were collectively defined as ASE genes (ASEGs). Among these ASEGs, 2499 showed consistent allelic bias across all five tissues and were classified as consistent ASEGs, whereas the remaining genes displayed tissue-specific or variable allelic expression patterns and were classified as inconsistent ASEGs (Table S10). Overall, consistent ASEGs exhibited greater allelic expression divergence than inconsistent ASEGs (Fig. 3A).

To examine selective pressures acting on protein-coding sequences, pairwise *K_a_*/*K_s_* ratios were calculated for all allelic gene pairs. The majority of ASEGs exhibited *K_a_*/*K_s_* ratios below 1, indicating predominant purifying selection (Fig. 3B; Table S10). Among the 9141 ASEGs, 247 genes (2.7%) showed *K_a_*/*K_s_* ratios > 1 (Table S10). After excluding genes with *K_s_* < 0.01 to minimize estimation artifacts, 42 ASEGs with *K_a_*/*K_s_* > 1 were retained as candidates potentially experiencing positive selection (Table S11). Most of these genes showed moderate *K_a_*/*K_s_* values ranging from 1.05 to 1.7, whereas a small subset exceeded 2.0, suggesting heterogeneous selective pressures among loci (Fig. 3C).

No significant differences in *K_a_*/*K_s_* ratios were detected between consistent and inconsistent ASEGs (Fig. 3D). Similarly, allelic expression divergence, measured as log₂ fold change (log₂FC), did not differ significantly between the two groups in flower buds, stems, roots, or seeds (*P* > 0.05; Fig. 3E–H). In contrast, a modest but significant difference was detected in leaves (*P* < 0.05; Fig. 3I). Although these differences were generally subtle, consistent ASEGs tended to exhibit greater allelic expression divergence, consistent with more stable allele-specific regulation across tissues.

Functional enrichment analysis revealed clear differences between the two ASEG categories. Inconsistent ASEGs were significantly enriched in pathways related to secondary metabolism, lipid and carbohydrate metabolism, membrane and cell-surface modification (Fig. 3J). These pathways are widely recognized as highly dynamic and context-dependent, with expression patterns that vary substantially across tissues and developmental stages [28]. In contrast, consistent ASEGs were predominantly associated with core cellular housekeeping processes, including central metabolism, cofactor biosynthesis, and cellular quality control (Fig. 3K). This functional partitioning supports distinct regulatory regimes, with inconsistent ASEGs subject to flexible, context-dependent allelic regulation, whereas consistent ASEGs maintain stable allele-specific expression across tissues.

Functional annotation of the 42 ASEGs with *K_a_*/*K_s_* > 1 revealed a nonrandom distribution across functional categories. A substantial proportion of these genes encoded disease resistance-related proteins, including NBS-LRR-type resistance proteins, receptor-like kinases, and transcriptional regulators such as NAC and LOB domain-containing proteins (Fig. 3L; Table S11). Examination of allelic expression patterns across tissues showed that several of these genes also exhibited pronounced regulatory divergence, with absolute log₂FC values exceeding 3 in one or more tissues. For example, pathogenesis-related protein 5 (*PR5*) and receptor-like protein 1 (*RLP1*) showed consistent allelic expression differences across tissues, whereas *RGA4* and *RGA3* displayed strong tissue-specific allelic divergence (Fig. 3L).

### Identification of sex-associated genomic regions based on coverage-based genome-wide association study (GWAS) and structural variation analyses

To identify sex-associated genomic regions and candidate sex-associated loci in *V. montana*, whole-genome resequencing data from 178 naturally grown individuals reported in our previous study [25], including 88 males and 90 females, were aligned to the female haplotype-resolved reference genome. The average mapping rate across all samples was 98.36%, indicating high-quality read alignment and suitability of the female reference for population-scale analyses (Table S12).

**Figure 4:**
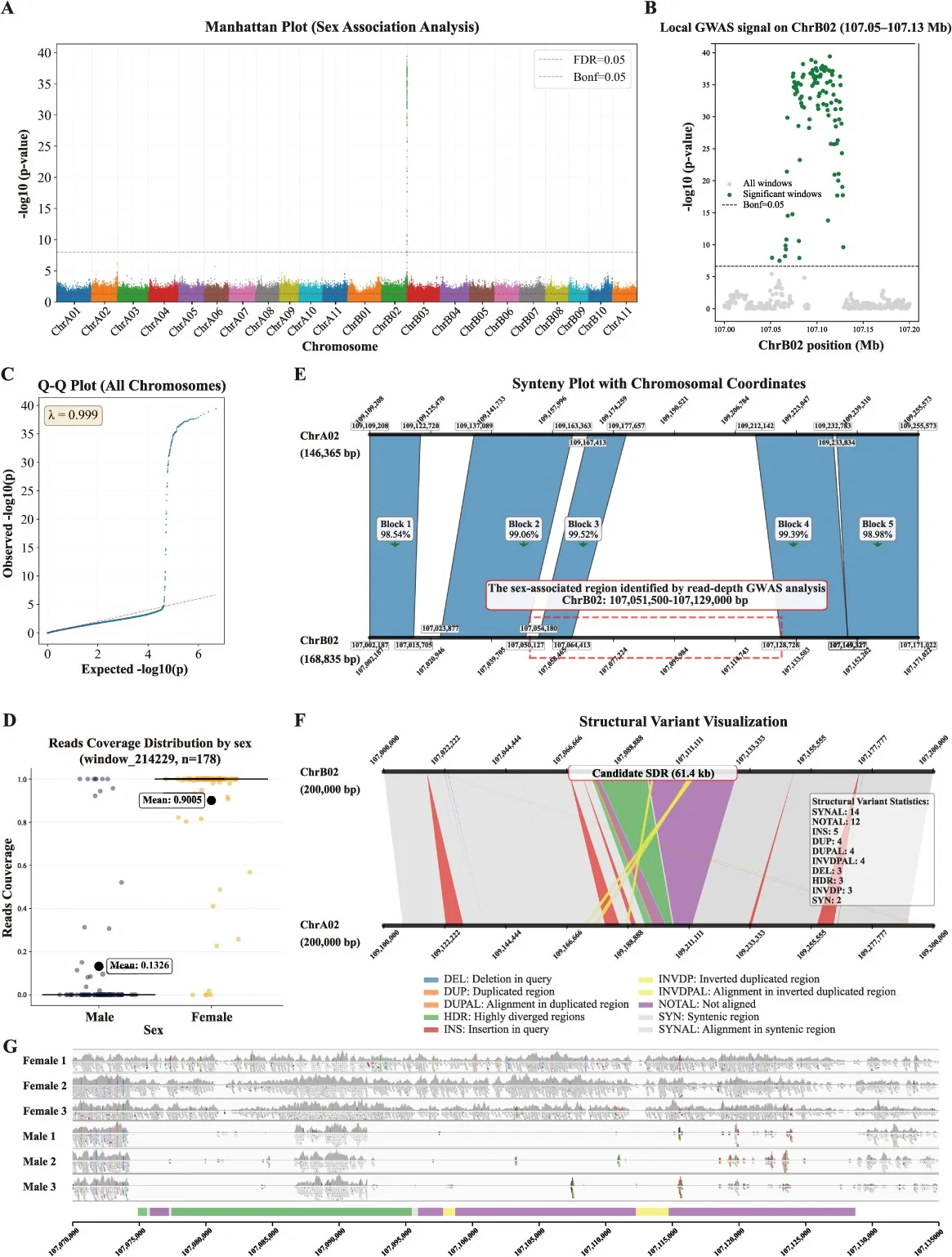
Identification of a candidate sex-determining region (SDR) on ChrB02 through integrated coverage-based GWAS and structural variation analyses. (A) Manhattan plot of the coverage-based GWAS results, showing genomic windows plotted by chromosomal position and statistical significance (−log10 *P*-value). Horizontal dashed lines indicate the Bonferroni- and FDR-corrected significance thresholds. (B) Enlarged view of GWAS signals on ChrB02 (107.0–107.2 Mb), with Bonferroni-significant windows highlighted. (C) Quantile–quantile (Q-Q) plot of observed versus expected −log10(*P*) values across all chromosomes, showing minimal genomic inflation (λ = 0.999). (D) Coverage-based distribution of representative sex-associated genomic windows, showing contrasting coverage patterns between male and female individuals. (E) Synteny plot illustrating local alignments between ChrB02 (107 002 187–107 171 022 bp) and its homologous haplotype ChrA02 (109 109 208–109 255 573 bp). The sex-associated interval identified by coverage-based GWAS is highlighted by a dashed box. (F) Structural variation landscape of the candidate region, visualizing duplications, insertions, deletions, inversions, syntenic blocks, and not aligned (NOTAL) segments between ChrB02 and ChrA02. (G) IGV visualization of read alignments across representative NOTAL segments on ChrB02. Shown are read coverage tracks from three female and three male individuals aligned to the female haplotype-resolved reference genome. Most NOTAL segments display robust read support in female samples but markedly reduced or absent coverage in male samples, consistent with their ChrB02-specific (W-linked) nature.

Genome-wide read coverage was calculated using sliding windows of 1 kb with a 0.5-kb step size and subjected to coverage-based genome-wide association analysis, in which sex was treated as a binary grouping variable to identify windows showing significant coverage differences between males and females (Fig. 4A). Based on Welch’s *t*-test, a total of 110 genomic windows were identified as significantly associated with sex (*P* < 0.05; Table S13). Notably, all significant windows were tightly clustered, though not strictly contiguous, within a narrow interval of ~77.5 kb on ChrB02 (ChrB02: 107 051 500–107 129 000 bp; Fig. 4B). The association signal was exceptionally strong, with adjusted *P*-values ranging from 8.25 × 10^−35^ to 6.26 × 10^−3^ (Fig. 4B; Table S13). Quantile–quantile (Q-Q) plots generated across all chromosomes showed a pronounced deviation from the expected null distribution only at the extreme tail of small *P*-values, while the genomic inflation factor remained close to unity (λ = 0.999), indicating minimal systematic bias and a low false-positive rate (Fig. 4C). Together, these results demonstrate a robust and highly localized sex-associated signal on ChrB02.

Although sample sex was determined by phenotypic observation (Table S12), a small subset of individuals exhibited read coverage patterns within the sex-associated windows that were inconsistent with their recorded sex (Fig. 4D and Fig.  S3). Importantly, these discordant individuals showed coherent deviations across multiple adjacent windows, resulting in consistently elevated Z-scores throughout the region (Table S14), rather than isolated outlier windows. In contrast, the vast majority of samples displayed stable and directionally consistent coverage differences between males and females across all significant windows, supporting the overall reliability of the identified sex-associated interval (Fig. 4D and Fig.  S3).

To investigate the genomic basis underlying this localized association, whole-chromosome intragenomic alignment was performed between ChrB02 and its homologous haplotype ChrA02. The GWAS-defined interval on ChrB02 exhibited little to no detectable collinearity with ChrA02, whereas the immediately flanking regions remained highly conserved (Fig. 4E). Specifically, the upstream region (ChrB02: 107 023 877–107 050 127) aligned to ChrA02: 109137089–109 163 363 with 99.06% identity, and the downstream region (ChrB02: 107 128 728–107 149 327) aligned to ChrA02: 109 212 142–109 232 783 with 99.39% identity. Within the broader interval, only a short internal segment (ChrB02: 107 054 180–107 064 413) retained limited but high-identity alignment to ChrA02 (ChrA02: 109 167 413–109 177 657; 99.52%), indicating pronounced structural divergence within the sex-associated interval despite strong conservation of the surrounding chromosomal context.

To further resolve the structural basis of this localized loss of collinearity, we conducted fine-scale structural variation analysis using SyRI on a focused genomic region encompassing the GWAS-significant windows and adjacent collinear segments. This analysis revealed that the sex-associated interval comprises a complex mosaic of structural features rather than a single continuous rearrangement (Fig. 4F; Tables S15 and S16). Multiple short ChrA02-derived segments were duplicated on ChrB02, generating localized copy number variation events annotated as duplicated region (DUP) and alignment in DUP (DUPAL). These duplicated segments were interspersed with fragmented syntenic blocks (SYN and SYNAL) that retained partial collinearity but were frequently interrupted by highly diverged regions (HDRs), indicating substantial sequence divergence within otherwise homologous regions.

A localized duplication event was identified in which a single 228 bp segment on ChrB02 (107 075 523–107 075 750) corresponded to two distinct 227 bp homologous segments on ChrA02, forming a one-to-two alignment relationship (Table S17). SyRI classified this configuration as a copy gain (DUP), indicating localized copy number expansion between the two haplotypes (orange blocks in Fig. 4F; Table S17). Several inversion-associated copy-loss events (INVDP) were also detected within the sex-associated interval, spanning 878–1353 bp and further contributing to local structural divergence (yellow blocks in Fig. 4F; Table S17). Notably, one such event (INVDP8B) corresponds to an inversion-associated duplication structure involving an inverted-repeat pair (Fig. S4; Table S17). The ChrB02-derived segment (IR-B1; ChrB02: 107 097 787–107 098 664 bp; 878 bp) harbors the previously validated female-specific InDel marker, whereas its ChrA02 counterpart (IR-A; ChrA02: 109 188 120–109 189 009 bp; 890 bp) carries the corresponding non-sex-specific genotype shared by both sexes (Table S18). IR-A and IR-B1 share 95.62% nucleotide identity and occur in opposite orientations, consistent with inversion-associated duplication. Interestingly, the sequence corresponding to IR-A on ChrA02 is embedded within a collinear block (SYNAL6B) shared between ChrA02 and ChrB02 (Fig. S4; Table S16). Comparative analysis of this syntenic region further revealed a second inverted copy on ChrB02 (IR-B2; ChrB02: 107 069 546–107 070 432; 887 bp), nearly identical to IR-A (98.76% identity) and arranged in inverted orientation relative to IR-B1 (Fig. S4; Table S18). Consequently, IR-B1 and IR-B2 form an additional inverted-repeat pair on ChrB02, sharing 95.95% identity.

In addition to duplications and inversions, multiple large insertion and deletion variants were detected throughout the interval (Fig. 4F). These long InDels were spatially interspersed with not aligned (NOTAL) segments, HDRs, and duplicated segments, further disrupting local collinearity between the two haplotypes. Moreover, multiple NOTAL segments were identified, corresponding to haplotype-specific sequences that could not be reliably aligned between ChrB02 and ChrA02 (Fig. 4F). These NOTAL segments accounted for a substantial proportion of the sex-associated interval and were distributed throughout the region rather than confined to a single continuous block.

To further illustrate the haplotype-specific nature of the NOTAL regions, read coverage patterns were visualized using Integrative Genomics Viewer (IGV) in representative male and female individuals. In most cases, NOTAL segments showed robust read support in female samples but were largely devoid of reads in males, consistent with their classification as haplotype-specific sequences on ChrB02 (Fig. 4G). Notably, a small subset of individuals exhibited read coverage patterns that were inconsistent with their recorded phenotypic sex, with some phenotypic females showing reduced coverage and occasional phenotypic males displaying detectable reads within the NOTAL regions (Fig. S5), consistent with the read coverage deviations observed in the GWAS analysis. Such discrepancies may reflect phenotyping uncertainty, local mapping ambiguity in highly repetitive regions, or structural polymorphism segregating within the population.

Together, these structural features explain the sharp contrast between the high conservation of flanking regions and the extensive divergence within the sex-associated interval on ChrB02, supporting the presence of a highly differentiated haplotype-specific region. We therefore define this region as a candidate W-specific SDR on ChrB02, spanning ~61.4 kb (ChrB02: 107 067 319–107 128 727 bp; Fig. 4F).

### Polymerase chain reaction (PCR) validation of female-specific sequences within the W-specific sex-determining region

**Figure 5:**
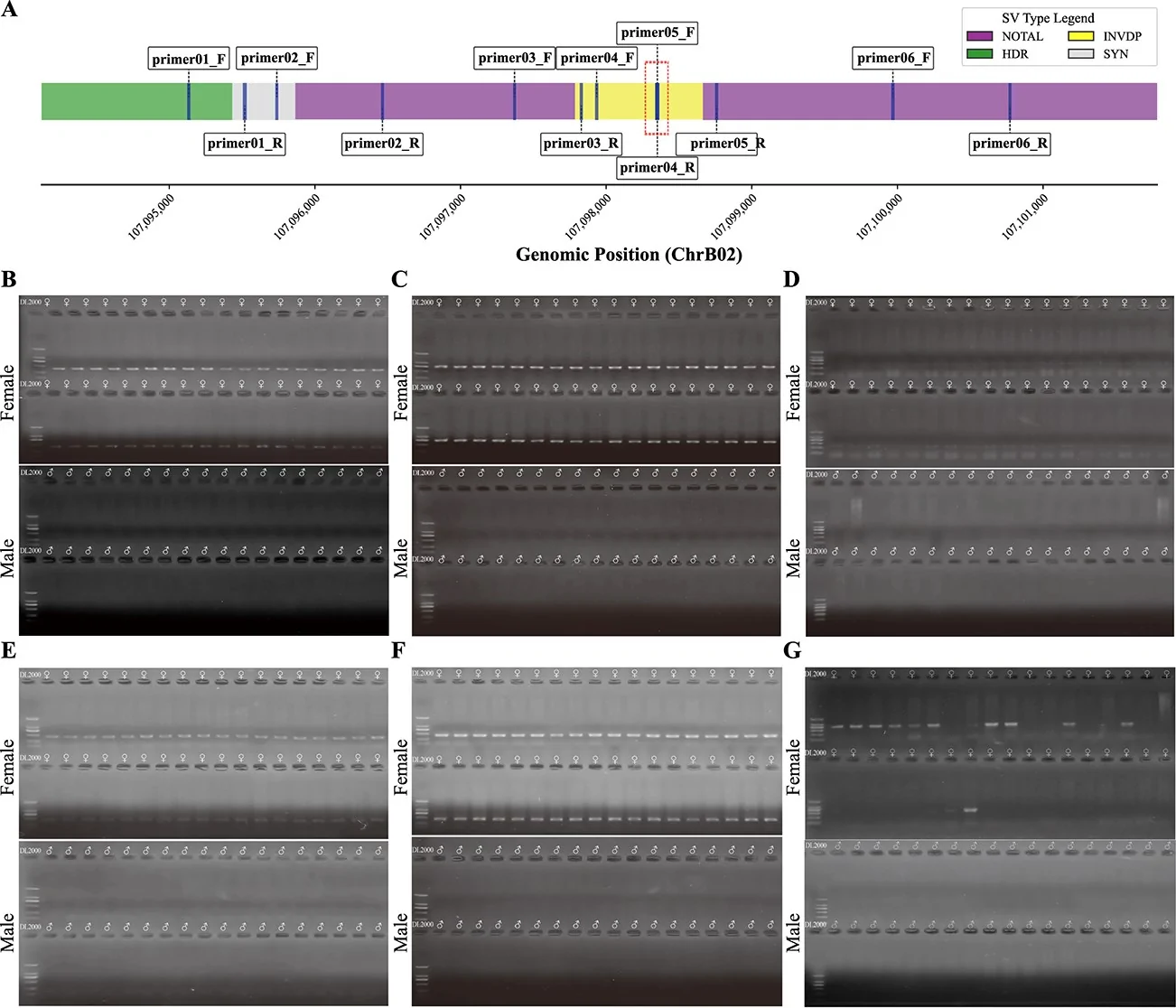
PCR validation of female-specific sequences within the candidate SDR on ChrB02. (A) Schematic representation of the candidate SDR showing the structural features inferred from SyRI analysis, including NOTAL, inverted duplicated regions (INVDP), highly diverged regions (HDR), and syntenic segments (SYN). The genomic positions of six primer pairs (primer01-primer06) are indicated. The dashed box highlights the primer pair targeting an inverted duplicated segment (IR-B1) that harbors a previously validated female-specific InDel marker. (B–F) Agarose gel electrophoresis results of PCR amplification using primer01 to primer05, respectively. Clear amplification products are consistently detected in all female samples, whereas no detectable bands are present in male samples, indicating female-specific amplification. (G) PCR amplification results obtained using primer06, which does not exhibit a consistent sex-specific amplification pattern. For all gels, a DL2000 DNA ladder (100, 250, 500, 750, 1000, and 2000 bp) was used as a molecular size marker. Female and male samples were derived from individuals whose phenotypic sex was fully concordant with coverage-based GWAS results.

To experimentally validate the female specificity of sequences within the W-specific SDR, six PCR primer pairs were designed to target representative NOTAL segments and adjacent structurally complex regions (Fig. 5A; Table S19). One target region corresponded to the 878-bp inverted duplicated segment IR-B1, which harbors the previously validated female-specific InDel marker. Accordingly, the downstream primer of primer04 and the upstream primer of primer05 were positioned within this marker-containing sequence, enabling direct assessment of its female-specific presence.

PCR assays were performed using a subset of individuals from the GWAS population. Specifically, 36 females and 36 males were randomly selected from samples whose phenotypic sex was fully concordant with the coverage-based GWAS results (Table S20), thereby minimizing potential ambiguity arising from coverage-discordant individuals. Among the six primer pairs tested (Table S19), five consistently produced clear amplification products in all female samples but showed no detectable amplification in male samples (Fig. 5B–F), supporting the presence of multiple female-specific genomic segments within the SDR. In contrast, one primer pair did not exhibit a fully consistent sex-specific amplification pattern (Fig. 5G), likely reflecting local sequence heterogeneity or structural complexity associated with inverted duplication, breakpoint-adjacent regions, or locally elevated guanine-cytosine (GC) content.

Overall, these PCR results provide independent experimental evidence that the W-specific SDR contains multiple female-specific sequences embedded within a structurally heterogeneous genomic landscape.

### Identification and functional validation of candidate sex-associated genes within the W-specific sex-determining region

**Figure 6:**
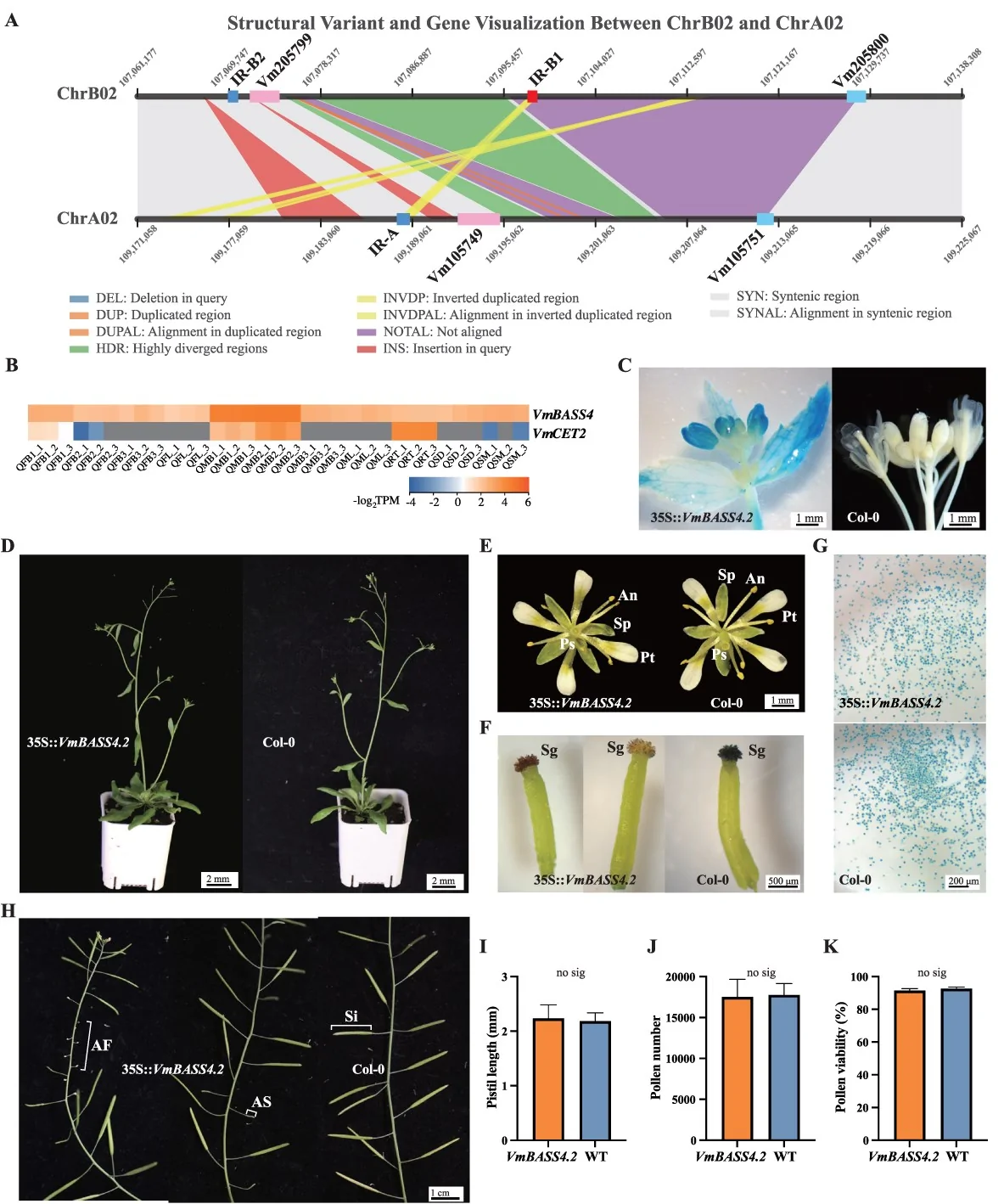
Candidate gene identification at the SDR boundaries and functional assessment of *VmBASS4*. (A) Structural variant landscape of the SDR between ChrB02 and ChrA02. Two protein-coding genes flank the SDR boundaries on ChrB02: *Vm205799* (*VmBASS4.2*) at the 5′ end and *Vm205800* (*VmCET2.2*) at the 3′ end, with their ChrA02-linked homologous alleles *Vm105749* (*VmBASS4.1*) and *Vm105751* (*VmCET2.1*) indicated. The inverted repeat elements IR-B2, IR-B1, and IR-A are labeled, highlighting their asymmetric positioning relative to *VmBASS4* across the two haplotypes. (B) Heatmap of gene-pair expression levels (log_2_TPM), calculated as the combined TPM of both alleles, for *VmBASS4* (*Vm205799*/*Vm105749*) and *VmCET2* (*Vm205800*/*Vm105751*) across vegetative tissues (roots, stems, leaves, seeds) and developing flower buds of both sexes. QFB1–3 represent female flower primordia, early, and middle developmental stages; QMB1–3 represent male flower primordia, early, and middle stages. (C) Representative floral phenotype of *A. thaliana* plants overexpressing *VmBASS4.2* under the CaMV 35S promoter (35S::*VmBASS4.2*) compared with wild-type Col-0 (Columbia ecotype). (D) Whole-plant morphology of 35S::*VmBASS4.2* transgenic lines and Col-0 grown under identical conditions, showing no significant differences in flowering time or overall growth architecture. (E) Representative mature flowers of 35S::*VmBASS4.2* and Col-0. Floral organs are labeled: sepals (Sp), petals (Pt), anthers (An), and pistil (Ps). (F) Close-up view of pistils, showing reduced stigma (Sg) receptivity in a subset of 35S::*VmBASS4.2* flowers. (G) Pollen viability assay of 35S::*VmBASS4.2* and Col-0, indicating comparable pollen viability between genotypes. (H) Representative inflorescences showing partial floral abortion and aborted siliques in 35S::*VmBASS4.2* plants compared with normal silique development in Col-0. AF, abortive flower; AS, abortive siliques; Si, siliques. (I–K) Quantification of pistil length (I), pollen number per 100 flowers (J), and pollen viability percentage (K) in 35S::*VmBASS4.2* and Col-0 flowers. Data in (I–K) are shown as mean ± SD; ‘no sig’ indicates no statistically significant difference between transgenic and wild-type plants.

Only two protein-coding genes were identified at the boundaries of the SDR: *Vm205799* at the 5′ boundary and *Vm205800* at the 3′ boundary, each with a single homologous allele on ChrA02 (*Vm105749* and *Vm105751*, respectively; Fig. 6A). Notably, IR-A and IR-B2 are located in close proximity to *Vm105749* and *Vm205799*, respectively. Specifically, IR-A lies 3034 bp upstream of *Vm105749* on ChrA02, whereas on ChrB02, IR-B2 is positioned 1272 bp upstream and IR-B1 occurs 23 328 bp downstream of *Vm205799* (Fig. 6A). This asymmetric repeat arrangement indicates pronounced divergence in the local regulatory landscape between haplotypes.

Comparative sequence analysis showed that *Vm205799* and *Vm105749* encode nearly identical proteins, differing only by a single-amino-acid truncation at the C terminus caused by a premature stop codon (Fig. S6A), making substantial protein-level functional divergence unlikely. Similarly, *Vm205800* and *Vm105751* encode identical amino-acid sequences (Fig. S6B). Functional annotation revealed that *Vm205799*/*Vm105749* correspond to homologs of the sodium/metabolite cotransporter BASS4 (hereafter *VmBASS4.2*/*VmBASS4.1*), whereas *Vm205800*/*Vm105751* correspond to homologs of CENTRORADIALIS-like 2 (*VmCET2.2*/*VmCET2.1*).

Because of the extremely high sequence similarity between allelic pairs, together with the structural complexity of the SDR, precluded reliable allele-specific expression quantification, transcript abundance was summarized at the gene-pair level by combining Transcripts Per Million (TPM) values from both alleles. *VmBASS4* exhibited broad expression across all examined tissues, including roots, stems, leaves, seeds, and developing flower buds of both sexes (Fig. 6B). Notably, *VmBASS4* expression was markedly elevated in male reproductive tissues, peaking in male flower primordia (QMB1; ~22 TPM) and remaining high during early stamen development (QMB2; ~23 TPM; Fig. 6B). In contrast, *VmCET2* displayed a more restricted and stage-specific expression pattern. Detectable transcripts were primarily observed in floral primordia and early reproductive stages, including female flower primordia (QFB1), male flower primordia (QMB1), and early stamen development (QMB2), as well as in roots (QRT; Fig. 6B). *VmCET2* expression was highest in roots (~17 TPM) and moderate during early male floral development (QMB2; ~10–14 TPM) but was largely absent from leaves, stems, seeds, and later floral stages (Fig. 6B).

Given its broad reproductive expression dynamics and close proximity to major structural rearrangements at the SDR boundary, *VmBASS4* was prioritized for functional validation, whereas *VmCET2* was not pursued further in heterologous assays. To test whether altered *VmBASS4* expression can influence reproductive development, we generated *A. thaliana* transgenic plants overexpressing *VmBASS4.2* under the CaMV 35S promoter (35S::*VmBASS4.2*; Fig. 6C). Overall plant architecture and flowering time of the transgenic lines were indistinguishable from those of wild-type Col-0 (Columbia ecotype) plants (Fig. 6D), and floral organ morphology was largely unaffected (Fig. 6E). No significant differences were observed in pistil length, pollen number, or pollen viability (Fig. 6G, I–K). However, a subset of transgenic flowers exhibited reduced stigma receptivity (Fig. 6F), accompanied by partial floral abortion and aborted siliques (Fig. 6H). Together, these results suggest that disruption of *VmBASS4.2* expression levels can compromise reproductive success, supporting its potential involvement in regulatory remodeling associated with SDR evolution.

## Discussion

### Resolving allelic heterogeneity through haplotype-resolved genome assembly

Conventional hybrid assemblies typically collapse the two haplotypes of a diploid genome into a single consensus sequence, thereby masking allelic heterogeneity and obscuring biologically meaningful variation such as single-nucleotide polymorphisms, small insertions/deletions, and structural variants [29, 30]. This limitation is particularly pronounced in highly heterozygous plant genomes, where sequence divergence between homologous chromosomes can lead to chimeric assemblies, allelic misassignment, and inaccurate gene models [31]. In contrast, haplotype-resolved genome assembly enables the independent reconstruction of homologous chromosomes, allowing accurate phasing of heterozygous loci and preserving allelic integrity across the genome [32].

In recent years, chromosome-scale reference genomes have been reported for several Euphorbiaceae species, including *Manihot esculenta* [33], *Hevea brasiliensis* [34], *Ricinus communis* [35], *Jatropha curca* [36], *V. fordii* [37], *V. montana* [25], and *Mercurialis annua* [38]. While these assemblies have substantially advanced genomic resources for the family, they are haploid-collapsed representations and therefore provide limited resolution of allele-specific variation. In this context, the female *V. montana* genome presented here constitutes, to our knowledge, the first chromosome-level, haplotype-resolved reference genome within the Euphorbiaceae. This resource provides a critical foundation for the precise localization of SDRs, the dissection of *cis*-regulatory divergence, and the functional interpretation of allelic variation underlying complex traits.

### Distinct evolutionary and regulatory patterns underlying allele-specific expression in *V. montana*

Through a systematic analysis of allelic imbalance in *V. montana*, we detected pervasive ASE across multiple tissues, with a subset of genes exhibiting consistent allelic bias. Although consistent ASEGs showed greater expression divergence than inconsistent ASEGs, their *K_a_*/*K_s_* ratios were not significantly higher. This pattern suggests that purifying selection continues to strongly constrain coding-sequence evolution in genes with essential cellular functions, whereas divergence in gene regulation may constitute a more prominent and flexible evolutionary route for allelic differentiation [39, 40].

The pronounced functional dichotomy between consistent and inconsistent ASEGs suggests the presence of two contrasting regulatory modes in *V. montana*. Consistent ASEGs, enriched in core housekeeping processes, likely operate under a stable regime. In contrast, inconsistent ASEGs are associated with plastic pathways and exhibit a flexible regulatory regime, whereby their tissue-dependent allelic switching provides rapid, condition-specific tuning of gene expression [41, 42]. This observation aligns with the established view that such context-dependent ASE enhances phenotypic plasticity and adaptive potential [43].

Notably, a small subset of ASEGs exhibited signatures of positive selection and was significantly enriched in disease resistance functions. The coupling of positive selection signals with allele-specific expression in genes such as *PR5* and *RLP1* suggests that both protein sequence evolution and regulatory divergence may be simultaneously targeted by selection, potentially leading to novel resistance specificities or expression dynamics [44–46]. Given the strong resistance of *V. montana* to fusarium wilt compared with *V. fordii* [26], the identification of candidate positively selected resistance alleles provides new clues for deciphering the underlying molecular mechanisms in this species.

### Sex-determining region evolution and regulatory remodeling

In most plant lineages, sex chromosomes remain at an incipient stage of differentiation, with sex-specific loci confined to small SDRs rather than spanning fully heteromorphic chromosomes [2, 5]. Here, coverage-based GWASs identified a highly localized sex-associated signal on ChrB02, with all significant windows clustered within a narrow interval (~77.5 kb). Such a compact footprint is typical of young and largely homomorphic sex chromosome systems, in which recombination suppression is restricted to a limited W-specific region while flanking sequences retain autosome-like collinearity, as reported in *Actinidia* and *Ficus* [7, 47, 48]. Although the GWAS-defined interval includes both the diverged region and adjacent conserved segments, fine-scale structural analysis resolved a smaller core SDR spanning ~61.4 kb on ChrB02. This core region does not represent a single continuous rearrangement, but rather a mosaic of microstructural variants, including duplications (DUP/DUPAL), fragmented syntenic blocks (SYN/SYNAL), and haplotype-specific segments (NOTAL). The interspersed architecture suggests an evolutionary history marked by repeated local duplication, divergence, and structural turnover, collectively explaining the pronounced haplotype divergence within an otherwise collinear chromosomal context [24].

A particularly informative feature is the three-copy inverted-repeat system (IR-A/IR-B1/IR-B2), which harbors the previously validated female-linked InDel marker [25] within the SDR on ChrB02 (IR-B1), with two homologous counterparts on ChrA02 (IR-A) and ChrB02 (IR-B2), respectively. This configuration indicates that sex-linked polymorphism in *V. montana* did not arise solely through gradual point mutation but instead emerged within a structurally dynamic genomic environment shaped by inversion-associated duplication [49]. Inverted repeats are known to promote nonallelic homologous recombination, replication slippage, and secondary-structure-mediated instability, thereby generating copy-number variation, inversions, and insertion/deletion turnover [50, 51]. Accordingly, the localization of a female-specific marker within NOTAL-flanked inverted repeats suggests that repeat-mediated remodeling may have contributed to the formation of a local recombination-suppressed microenvironment, enabling the accumulation of W-linked sequence differentiation without large-scale karyotypic divergence [52].

Notably, a minority of individuals displayed coverage-based patterns within NOTAL segments that were inconsistent with their recorded phenotypic sex. Because these deviations were coherent across multiple adjacent windows, they are unlikely to represent random noise [53]. Several nonmutually exclusive explanations may account for these discordances, including imperfect phenotypic sex assignment in long-lived woody perennials, structural or copy-number polymorphisms segregating within natural populations, and occasional mapping ambiguity in highly repetitive SDR sequences [54–56]. PCR validation further supported the presence of multiple female-specific segments within the SDR, as five of six primer pairs consistently amplified only in females. The failure of one primer pair likely reflects extreme breakpoint-adjacent heterogeneity within inverted duplications and high-divergence segments rather than the absence of female-specific sequence.

Interestingly, unlike systems governed by lineage-specific master regulators, the *V. montana* W-specific SDR does not harbor an obvious protein-coding gene showing strong sex-specific presence/absence or rapid coding divergence [2]. Instead, two conserved genes (*VmBASS4.2* and *VmCET2.2*) flank the SDR boundaries and retain highly similar allelic counterparts on ChrA02. *VmBASS4.2* differs from *VmBASS4.1* only by a single C-terminal truncation, arguing against neofunctionalization via protein innovation [15]. Rather, the pronounced asymmetry in local regulatory architecture, where inverted repeats and NOTAL segments lie in close proximity to *VmBASS4.2* on ChrB02 but not on ChrA02, supports a structural-first model. In this model, structural variation, particularly inverted-repeat-mediated structural remodeling, precedes and drives regulatory divergence without requiring the emergence of novel protein-coding genes. Accordingly, SDR evolution primarily reshapes *cis*-regulatory landscapes rather than generating new sex-determining proteins [57].

Expression profiling further links these boundary genes to the strongly dimorphic reproductive schedule of *V. montana*. Male inflorescences differentiate earlier and produce abundant floral primordia, whereas females initiate development later with fewer flowers per inflorescence [27]. Consistent with this heterochronic divergence, *VmBASS4* is strongly upregulated in early male flower primordia and stamen development, coinciding with the initial divergence of sex-specific pathways. This supports a role in modulating developmental timing or reproductive intensity, rather than functioning as a binary sex switch.

*Arabidopsis* functional assays further indicate that *VmBASS4.2* is a dosage-sensitive regulator of reproductive success, as its misexpression can compromise fertility. However, such pleiotropic effects do not establish it as a primary sex-determining gene. Instead, they indicate that *VmBASS4.2* is a developmentally responsive locus requiring precise expression control within the reproductive context. Consequently, we propose that during SDR evolution, structural rearrangements, not protein-coding changes, remodeled the sex-biased or heterochronic expression of *VmBASS4.2*. Such regulatory divergence acting on a dosage-sensitive gene may facilitate the stabilization of dioecy without the emergence of a novel master sex-determining switch.

### Limitations and future directions

Despite the strong population-level association and structural resolution of the W-specific SDR, several limitations remain. First, the SDR is enriched in repetitive and haplotype-specific sequences, and short-read resequencing may introduce mapping ambiguity, potentially contributing to the small fraction of sex-discordant individuals. Second, although *VmBASS4.2* is located at the SDR boundary and shows dosage-sensitive effects in heterologous assays, the current data do not establish it as a primary sex-determining factor, and additional W-linked regulatory elements or noncaptured transcripts within NOTAL segments may play causal roles. Third, the regulatory mechanisms underlying SDR-driven differentiation, including DNA methylation, chromatin accessibility, and small interfering RNA (siRNA)-mediated silencing, remain unexplored in *V. montana*. Future work integrating long-read population sequencing, epigenomic profiling, and functional validation in the native species will be essential to pinpoint causal sex-determining components, resolve SDR structural polymorphism, and clarify how local genomic remodeling stabilizes dioecy and reproductive development.

## Materials and methods

### Sample preparation and genome sequencing

A female individual of *V. montana* (Accession No. HXX03) from the National Tung Tree Germplasm Conservation Repository, Yongshun, Hunan, China, was collected and sequenced. Genomic DNA was extracted using the DNeasy Plant DNA Extraction Mini Kit (QIAGEN). Eight micrograms of genomic DNA were sheared using g-Tubes (Covaris) and concentrated with AMPure PB magnetic beads. Each SMRTbell library was constructed using the PacBio SMRTbell express template prep kit 2.0. The constructed libraries were size-selected on a BluePippin™ system for molecules ≥11 kb, followed by primer annealing and the binding of SMRTbell templates to the polymerases with the DNA/Polymerase Binding Kit. Sequencing was carried out on the PacBio Sequel II platform for 30 h at Annoroad Gene Technology Company. The Hi-C library was constructed following the previously published methodology [58] and sequenced on a DNBseq-T7 platform at GrandOmics Gene Technology (https://www.grandomics.com/).

### Genome size estimation, genome assembly, and assessment

Genome size was estimated using a 17-bp k-mer distribution analysis with Jellyfish v2.0 (https://www.cbcb.umd.edu/software/jellyfish/) and the publicly available PERL script estimated_genome_size.pl (https://github.com/josephryan/estimate_genome_size.pl). Hifiasm (0.16.1-r375) [30] was employed with a combination of HiFi and Hi-C reads in integrated mode to generate a set of two haplotype-resolved, phased contig-level assemblies. 3D-DNA [59] was used to scaffold the phased contigs into chromosomes. Finally, manual correction was performed using Juicebox v2.20.00 [60]. The Hi-C interaction matrix of HA and HB was created by HiCExplorer v3.7.3 [61]. The accuracy and completeness of these two haplotype genomes were assessed using BUSCO v5.7.1 [62] with the embryophyte_odb10 dataset of 1614 genes, LTR_retriever v2.9.8 [63], and k-mer-based assembly tool of Merqury v1.3 [64] following recommended parameters.

### Genome annotation

The Extensive de novo TE Annotator (EDTA v1.9.4) [65] was used to conduct whole genome *de novo* TE annotation following the recommended parameters. Following the masking of repeat regions identified by EDTA, a multi-faceted prediction strategy including *de novo*, homology-based, and RNA-seq-based gene prediction was employed to generate high-quality protein-coding genes in the *V. montana* genome assembly. Genomes of *A. thaliana* [66], *Oryza sativa* [67], *V. montana* [25], *V. fordii* [37]*, M. esculenta* [33], *J. curcas* [36], and *R. communis* [35] were searched for homology-based prediction using GeMoMa v1.8 [68]. Augustus v3.5.0 [69], GlimmerHMM v3.0.2 [70], and GeneMark-ES [71] were used for *de novo* gene model prediction. Second- and third-generation transcriptome sequencing data of different tissues were employed for transcript evidence, following the methods documented previously [25]. Finally, the gene sets predicted by *de novo*, homology-based, and RNA-seq-based gene prediction were integrated into a non-redundant and more comprehensive gene set by EVidenceModeler v2.1.0 [72] with manual curation. SwissProt, TrEMBL, Kyoto Encyclopedia of Genes and Genomes (KEGG), InterPro, National Center for Biotechnology Information (NCBI) non-redundant (Nr), and Gene Ontology (GO) [73] databases were integrated to gain functional annotation of protein-coding genes. Quality assessment of annotated gene sets was performed using BUSCO v5.7.1 [62].

tRNAs were identified using tRNAscan-SE v2.0.12 [74] with default parameters, rRNAs with Barrnap v0.9, and miRNAs/snoRNAs via infernal v1.1.5 [75] cmscan (RFAM 10 database) with recommended parameters.

### Comparative synteny analysis

JCVI (python version of MCScanX; v1.5.7) was employed to perform comparative synteny analysis between female haplotypes and the male genome with the parameters recommended by the authors [76].

### RNA-sequencing and gene expression analysis

Total RNA was extracted from roots, stems, leaves, flower buds, and seeds using the RNAprep Pure Plant Plus Kit (Polysaccharides & Polyphenolics-rich; TIANGEN), following the manufacturer’s protocol. All libraries were constructed and sequenced on the DNBSEQ-T7 platform by Guangzhou Gene Denovo Biotechnology Co. Ltd. Raw transcriptome reads were quality-filtered using fastp v1.0.1 [77] to generate clean data. Clean reads were first aligned to a Ribosome database using Bowtie2 v2.5.4 to identify and remove rRNA sequences. The remaining unmapped reads were then aligned to the reference genome using HISAT2 v2.2.1 [78]. Gene expression was quantified using Stringtie v3.0.0 [78] and normalized using the TPM method.

### Allelic gene characterization, expression, and enrichment analysis

Allelic genes were identified following the published method described in a previous study [79]. MCScanx v1.0.0 [80] was used to identify collinearity block gene pairs between two allelic chromosome pairs. Then, 1000 bp upstream and downstream of these gene pairs were compared using BLASTN. Finally, gene pairs with coding sequence alignment values >85% (evalue <1e-10) were identified as allelic genes. The *K_a_*, *K_s_*, and *K_a_*/*K_s_* values between alleles were computed using KaKs_Calculator v3.0 [81]. Differential expression analysis between alleles was performed using the R package DESeq2. KEGG enrichment analyses were conducted via the OmicShare Tools platform (https://www.omicshare.com).

To assess whether there were significant differences in the expression levels between consistent and inconsistent ASEGs within each tissue, a two-sample unpaired *t*-test was performed. The analysis compared the log_2_FC of expression for each group of ASEGs across the same tissue. Genes classified as consistent ASEGs were defined as showing consistent allele-specific expression across all five tissues, while genes classified as inconsistent ASEGs exhibited variable or tissue-specific allelic expression patterns. For each tissue, the mean log_2_FC for the two groups was compared, and statistical significance was determined using the unpaired t-test.

### Genome-wide association analysis

Genome resequencing data from 80 male and 90 female individuals (Table S14) were retrieved from the Genome Sequence Archive (GSA, https://ngdc.cncb.ac.cn/gsa) under accession number CRA009450 [25]. Paired-end resequencing reads were individually aligned to the female haplotype reference genome following previously described procedures. Briefly, read alignment was performed using the BWA-MEM algorithm implemented in BWA v0.7.19. The resulting alignments were sorted using SAMtools v1.21 [82], and PCR duplicates were identified and removed with the MarkDuplicates module of Picard Tools v3.4, yielding coordinate-sorted Binary Alignment/Map (BAM) files.

Based on the deduplicated BAM files, genome-wide read coverage was calculated using BEDTools v2.31.1 [83] with a sliding-window approach, employing 1-kb windows with a 0.5-kb step size. Sex was treated as a binary grouping variable, and window-wise association analyses were conducted by comparing read coverage values between male and female samples for each window. To quantify sample-specific deviations in read coverage within sex-associated genomic windows, Z-scores were calculated for each sample at each window. Positive and negative Z-scores indicate higher or lower coverage relative to the population mean for a given window, respectively. Z-scores were used to assess the direction and magnitude of sex-specific coverage differences at the individual level and to identify samples showing atypical coverage patterns within the sex-associated region.

Three statistical models were applied independently: Welch’s *t*-test, linear regression, and logistic regression. To control false-positive associations arising from multiple testing, both the Benjamini–Hochberg (BH) false discovery rate (FDR) correction and the Bonferroni correction were applied. Genomic windows significantly associated with sex were identified using a *P*-value threshold of <0.05 based on the Welch’s *t*-test results. The Bonferroni-corrected significance threshold was calculated as 0.05 divided by the total number of tested windows, and the corresponding −log10(*P*) value was used as the significance cutoff in Manhattan plots.

To characterize sample-level deviations within the sex-associated region, we focused on the 10 most statistically significant genomic windows identified by the Welch’s *t*-test after Bonferroni correction. These windows represent the strongest and most stable association signals and were used for individual-level assessment rather than for additional hypothesis testing. For each of the selected windows, the read coverage values were standardized across all samples by calculating Z-scores. For each individual, Z-scores were examined across the 10 windows to evaluate the direction and magnitude of coverage deviation relative to the population mean. Samples exhibiting Z-score directions consistently opposite to the expected sex-specific pattern across multiple windows were classified as showing discordant coverage signals.

All GWAS analyses were implemented in Python v3.11.13, with key dependencies including pandas for data handling, numpy for numerical computation, scipy.stats for statistical testing, statsmodels for regression analyses, and matplotlib and seaborn for data visualization.

### Structural variation analysis of the sex-associated region

To investigate structural variation underlying the sex-associated region on chromosome ChrB02, a two-step assembly-based comparative strategy was applied between the two female haplotypes. As an initial assessment of large-scale collinearity between the two haplotypes, ChrB02 from the HB assembly was aligned to its homologous chromosome ChrA02 from the HA assembly using MUMmer v3.23 (nucmer) with default parameters [84]. The resulting delta files were filtered using delta-filter with the parameters -l 1000, retaining only one-to-one alignments with a minimum alignment length of 1 kb. This preliminary alignment was used to identify broad patterns of collinearity, alignment gaps, and potential regions of structural divergence between ChrB02 and ChrA02.

Based on the coverage-based GWAS results and the preliminary alignment, a localized region spanning ChrB02: 107 000 000–107 200 000 bp was extracted from the HB assembly. This interval fully encompassed all genomic windows significantly associated with sex, as well as their immediate flanking collinear regions. The corresponding homologous region on ChrA02 (ChrA02: 109 100 000–109 300 000 bp) was extracted from the HA assembly to ensure complete coverage of potential alignment breakpoints and rearranged segments.

To comprehensively capture haplotype-specific structural variation and minimize potential directional bias in alignment, local sequence alignments were performed in a bidirectional manner, with ChrB02 aligned to ChrA02 (Table S15) and ChrA02 aligned to ChrB02 (Table S16) independently. Alignments were generated using nucmer with parameters --maxmatch -l 20 -c 50. The resulting alignments from both directions were subsequently merged and filtered using delta-filter with parameters -i 85 -l 50, retaining uniquely mapped alignments with a minimum sequence identity of 85% and a minimum alignment length of 50 bp. These filtered alignments were then used as input for SyRI to identify syntenic blocks and classify structural variants [85].

Structural variants, including insertions, deletions, inversions, tandem duplications, and translocations, were subsequently identified from the filtered local alignments using SyRI (v1.7.1; --allow-offset 5000, --maxsize 1 000 000) were restricted to the locally aligned regions and classified according to SyRI’s default structural variant categories. Only variants fully or partially overlapping the sex-associated interval on ChrB02 were retained for downstream analyses. Coordinates of the detected variants were integrated with the GWAS-significant windows to assess their spatial correspondence.

### Visualization of read coverage across NOTAL regions

To examine read coverage patterns across NOTAL regions and to assess their haplotype-specific nature, read alignments from representative male and female individuals were visualized using IGV (v2.19.5) [86]. BAM files generated from whole-genome resequencing were loaded alongside the haplotype-resolved female reference genome, and coverage tracks were displayed using default IGV settings with autoscaling enabled.

For visualization, three phenotypic female and three phenotypic male individuals were selected from the GWAS population. These individuals were chosen to represent typical coverage-based patterns observed at the population level while avoiding samples with globally abnormal sequencing depth. The inspected regions corresponded to NOTAL segments within the candidate sex-determining region on ChrB02, as defined by SyRI-based structural variation analysis. Read coverage was assessed qualitatively based on the presence or absence of aligned reads across NOTAL segments. In addition to representative individuals showing concordant sex-specific patterns, selected individuals exhibiting discordant read coverage signals in the GWAS analysis were visualized separately to evaluate potential sources of variation. The homologous region on ChrA02 was not visualized, as coverage-based GWAS and whole-genome alignment analyses indicated comparable coverage in both male and female individuals across this region, consistent with its autosomal and non-sex-specific nature.

### PCR validation of female-specific genomic segments

Based on the structural variation analysis of the candidate SDR on ChrB02, six primer pairs were designed to amplify representative NOTAL segments and adjacent structurally complex regions, including an INVDP harboring a previously validated female-specific InDel marker (Table S20). Primer design was performed using SnapGene (v4.3.6), with primer pairs selected to flank target regions while avoiding low-complexity sequences and minimizing potential secondary structures.

PCR validation was conducted using a subset of individuals from the GWAS population. Specifically, 36 female and 36 male individuals (Table S19) were randomly selected from samples whose phenotypic sex was fully concordant with the coverage-based GWAS results, thereby reducing potential ambiguity arising from coverage-discordant individuals (Table S18). Genomic DNA was extracted using a modified Cetyltrimethylammonium Bromide (CTAB) method and quantified prior to amplification.

PCR reactions were performed in a 10 μl reaction volume, containing ~25 ng genomic DNA, 0.2 μM of each primer, 5 μl of 2× PCR Master Mix, and nuclease-free water. Thermal cycling conditions consisted of an initial denaturation at 95°C for 3 min, followed by 30 cycles of denaturation at 95°C for 30 s, annealing at primer-specific temperatures for 30 s, and extension at 72°C for 30–60 s depending on amplicon length, with a final extension at 72°C for 5 min. PCR products were separated on 1% agarose gels and stained with GoldView™ nucleic acid dye for visualization under ultraviolet (UV) illumination. A DL2000 DNA ladder (100, 250, 500, 750, 1000, and 2000 bp) was used as the molecular size standard.

### Heterologous overexpression of *VmBASS4* and reproductive phenotypic assays in *A. thaliana*

To evaluate the functional consequences of altered *VmBASS4* expression on reproductive development, the coding sequence of the W-linked allele *VmBASS4.2* (*Vm205799*) was amplified and cloned into the binary vector pBWA(V)BS under the control of the CaMV 35S promoter, generating a 35S::*VmBASS4.2*-*GUS* overexpression construct. The recombinant plasmid was introduced into *A. thaliana* ecotype Columbia (Col-0) via *Agrobacterium tumefaciens*–mediated floral dip transformation. Transgenic plants were selected on MS medium supplemented with 20 mg/l Basta, and Basta-resistant T2 individuals were used for subsequent phenotypic analyses.

Transgene expression was verified by histochemical β-glucuronidase (GUS) staining using a commercial GUS staining kit (Coolaber, China) according to the manufacturer’s instructions. After staining, tissues were decolorized and examined under a stereomicroscope.

Reproductive phenotypes were assessed in both transgenic and wild-type plants. Pistil length was measured to assess potential effects on female organ development, with 20 pistils collected from independent flowers of 35S::*VmBASS4.2* lines and Col-0 plants, respectively, and lengths determined from stereomicroscope images. Stigma receptivity was evaluated using a benzidine-H₂O₂ staining assay, in which pistils were immersed in freshly prepared staining solution and receptive stigmas developed a characteristic blue-black coloration indicative of peroxidase activity.

Pollen number and viability were assessed using a benzidine–hydrogen peroxide staining method. For each genotype, 100 flowers at the white-bud stage were collected and immersed in 500 μl of 10% sucrose solution. Samples were shaken vigorously for 2 min to release pollen grains, after which floral tissues were removed, and the suspension was centrifuged at 12 000 rpm for 2 min. The pollen pellets were resuspended in 20 μl benzidine-H₂O₂ staining solution and incubated for 10 min. A 2 μl aliquot was examined under a stereomicroscope for imaging and counting. Blue-stained pollen grains were scored as viable, whereas unstained grains were considered nonviable. Total pollen number per 100 flowers and pollen viability percentages were calculated based on dilution factors. All assays were performed with three biological replicates for both transgenic and wild-type plants.

Quantitative comparisons of pistil length, pollen number, and pollen viability between 35S::*VmBASS4.2* transgenic lines and Col-0 plants were conducted using Student’s *t*-test.

## Supplementary Material

Web_Material_uhag149

## Data Availability

The PacBio sequencing data, Hi-C data, and RNA-seq data have been deposited to Genome Sequencing Archive (GSA) databases in the BIG Data Center (https://ngdc.cncb.ac.cn/gsa) under BioProject accession numbers PRJCA018999 and PRJCA045463. The ISO-seq data and resequencing short reads were downloaded from the GSA under BioProject accession numbers PRJCA009616 and PRJCA014217.
