# Supplementary material for: Haplotype-resolved genome assembly of female Vernicia montana elucidates its W-specific sex-determination region
Source: Hortic Res. 2026 Apr 16;13(9):uhag149. doi: 10.1093/hr/uhag149 (PMC13503967; doi:10.1093/hr/uhag149)
Supplement: Supplementary file 1 [file web_material_uhag149.zip › supplementary figures.doc]

Figure S1. 17-mer frequency distribution plot from genome survey sequencing of *Vernicia montana* (Accession No. HXX03), with estimated genome size and heterozygosity

Figure S2. Genome-wide distribution of LTR assembly index (LAI) for two haplotypes of the haplotype-resolved genome of *V. montana*

Figure S3. Reads coverage patterns of the top 2 to 10 sex-associated genomic windows (A-I: top2-top10 by significance). Each panel shows coverage values (y-axis) stratified by sex (x-axis: Male = navy blue, Female = orange) for 178 samples. Dots represent individual samples (60% transparency), boxplots show distribution (median = black line), and black circles indicate mean coverage (labeled with exact values). All windows displayed sex-specific coverage patterns, confirming sex association.

Figure S4. Inverted repeat architecture associated with the INVDP8B rearrangement in the sex-associated interval. IR-B1 is marked by a red star, whereas IR-A and IR-B2 are marked by blue stars. Arrows indicate the orientation of each repeat copy, highlighting the inverted arrangement among the three homologous segments. Colored blocks represent syntenic alignments and structural variant classes (NOTAL, SYN, INVDP) across the candidate sex-determining region.

Figure S5. Representative IGV snapshots illustrating atypical read coverage patterns within NOTAL regions for individuals whose read-depth profiles are inconsistent with their recorded phenotypic sex.

Figure S6. ClustalW alignment of paired homologous protein sequences. (A) Vm105749 vs. Vm205799; (B) Vm105751 vs. Vm205800. Asterisks indicate identical amino acid residues.
